# Editorial: Deciphering the immunological and neuronal regulators of diabesity

**DOI:** 10.3389/fendo.2025.1657868

**Published:** 2025-07-18

**Authors:** Chandan Sona, Rakesh B. Patel, Surendra Ugale, Dinesh Kumar Verma

**Affiliations:** ^1^ University of Miami, Miami, FL, United States; ^2^ Department of Internal Medicine, Division of Hematology, Oncology and Blood & Marrow Transplantation, University of Iowa, Iowa City, IA, United States; ^3^ Kirloskar Hospital, Hyderabad, India; ^4^ Delaware State University, Dover, DE, United States

**Keywords:** type-1 diabetes (T1D), type-2 diabetes (T2D), insulin resistance, diabetic retinopathy (DR), immune cell infiltration, autoantibody

Diabesity is a global epidemic and a complex disorder caused by a decrease in beta-cell function insulin and leptin resistance. Several factors contribute to the pathophysiology of this disease. To preserve endocrine function, it is critical to understand the cause and mechanisms of the progressive deterioration of the endocrine function and how non-endocrine factors like immune cells and neuronal cells regulate its function. Due to the disease’s complexity, the in-depth understanding of the mechanism leading to the pathophysiological conditions of diabesity is yet to be understood properly. The field of inter-organ interaction or cell-to-cell interaction studies or genetic factors has grown significantly over the past decade and shows many promising results of how inter-organ interaction or genetic factors influences cellular function and metabolic homeostasis. Therefore, to understand diabesity more mechanistically, we should understand how non-endocrine cell populations or other factors affect endocrine cell function.

In this Research Topic, we summarize findings from four compelling studies that exemplify how integrating Mendelian randomization (MR), transcriptomics, proteomics, and autoantibody profiling with clinical data is transforming our understanding of diabetes. These works provide a significant understanding of understanding complex disease like diabetes with interdisciplinary approach.

## Immune cells and type 1 diabetes: insights from mendelian randomization

The autoimmune nature of T1D has long been recognized, but dissecting the precise immune mechanisms driving β-cell destruction has proven challenging. The study by Yu et al. innovatively applies Mendelian Randomization (MR) to investigate the causal roles of specific immune cell subsets in the development of T1D. By using genetic variants as proxies for immune cell traits, the authors sidestep confounding factors that typically obscure observational studies, offering a more causally robust perspective. The study identified significant associations between T1D susceptibility and immune markers such as CD28 on CD28+CD45RA+CD8 T cells, and others including monocyte and dendritic cell surface markers. These findings suggest that dysregulated signaling and cellular composition within the immune system play a key role in the initiation and progression of T1D. Therapeutically, this provides a rationale for immune modulation approaches that precisely target these implicated subsets, potentially arresting autoimmune activity before irreversible β-cell damage occurs.

## Transcriptomic and Mendelian randomization integration in diabetic retinopathy

Diabetic retinopathy (DR) is one of the most common and devastating microvascular complications of diabetes, yet the molecular mechanisms that govern its onset and progression are not fully understood. Jiang and Han employ an integrative approach by combining transcriptomic profiling with Mendelian randomization to reveal genes associated with mitochondrial dysfunction and programmed cell death—two hallmarks of DR pathophysiology.

They examine 658 genes and associated 12 genes with DR. Due to their pronounced upregulation MYC and SLC7A11 were the two candidate genes pointed out by the authors. MYC, known for its role in cell proliferation and apoptosis, and SLC7A11, implicated in oxidative stress regulation, represent promising targets for therapeutic development. This study exemplifies the strength of combining large-scale gene expression data with MR to move beyond correlation and toward causation. It opens the door to gene-based interventions and highlights the potential of targeting mitochondrial pathways to halt or reverse vision-threatening complications.

## Proteomic insights into diabesity using *Caenorhabditis elegans*

In the context of T2D, the interrelationship between obesity and insulin resistance—commonly referred to as “diabesity”—has been extensively documented. Yet, unraveling the precise molecular interactions has been hindered by the complexity of human metabolic networks. Subhadra et al. take an innovative turn by employing the model organism *Caenorhabditis elegans* (C. elegans) to study the proteomic alterations associated with diabesity.

When exposed to glucose levels mimicking human hyperglycemia, *C. elegans* exhibited increased intracellular triglyceride accumulation, shortened lifespan, and reduced pharyngeal pumping—hallmarks of metabolic dysfunction. Proteomic analysis revealed changes in key protein networks that parallel those observed in humans, thereby validating the utility of this model organism. This study not only underscores the value of *C. elegans* in high-throughput screening and drug discovery but also provides foundational insights into conserved biological processes that drive diabesity. It paves the way for identifying novel metabolic regulators and therapeutic compounds in a cost-effective and ethically sound manner.

## Autoantibody profiling enhances diabetes classification

Accurate diagnosis remains a cornerstone of effective diabetes management. However, differentiating between T1D and T2D, especially in adult-onset cases with overlapping features, is a persistent clinical challenge. Xian et al. address this by integrating diabetes-specific autoantibody profiles with clinical and laboratory indices to enhance diagnostic precision.

Involving 522 diabetic patients divided into training and validation cohorts, the study identified predictors such as age, prealbumin (PA), high-density lipoprotein cholesterol (HDL-C), islet cell autoantibodies (ICA), islet antigen 2 autoantibodies (IA-2A), glutamic acid decarboxylase antibody (GADA), and C-peptide levels. This multi-parametric model significantly improved the ability to distinguish T1D from T2D. The implications are profound: earlier and more accurate classification enables tailored treatment regimens, reduces misdiagnosis, and ensures appropriate monitoring for complications.

## The road ahead

As diabetes prevalence continues to challenge health systems globally, interdisciplinary collaboration will be key to advancing research and improving outcomes. Such approaches hold immense promise. Bridging genetics, immunology, molecular biology, neuroscience and clinical practice is not only desirable—it is necessary to understand this complex disease. The studies reviewed here demonstrate the potential of such collaborations to produce findings that are both scientifically rigorous and clinically relevant.

In summary, these four studies exemplify the power of interdisciplinary approach to dissect and address the multifactorial nature of diabetes. They highlight important findings in the challenging field of diabetes research and contribute valuable knowledge for researchers working with similar approaches.

Diabetes is a complex disease, and understanding it requires an interdisciplinary approach—integrating genetics, proteomics, immunology, and clinical innovation. As the editor of this Research Topic, I find these articles particularly exciting. They represent significant progress toward better understanding, management, and ultimately, prevention of diabetes.

